# Motion and dosimetric criteria for selecting gating technique for apical lung lesions in magnetic resonance guided radiotherapy

**DOI:** 10.3389/fonc.2023.1280845

**Published:** 2023-11-23

**Authors:** Matteo Galetto, Matteo Nardini, Amedeo Capotosti, Guenda Meffe, Davide Cusumano, Luca Boldrini, Giuditta Chiloiro, Angela Romano, Claudio Votta, Maria A. Gambacorta, Luca Indovina, Lorenzo Placidi

**Affiliations:** ^1^ Radiotherapy Department, Università Cattolica del Sacro Cuore, Roma, Italy; ^2^ Radiotherapy Department, Fondazione Policlinico Universitario A. Gemelli IRCCS, Roma, Italy; ^3^ Unità Operativa Semplice (UOS), Fisica Medica – Mater Olbia Hospital, Olbia, Italy

**Keywords:** MRgRT, gating, target motion, SBRT, lung

## Abstract

**Introduction:**

Patients treatment compliance increases during free-breathing (FB) treatment, taking generally less time and fatigue with respect to deep inspiration breath-hold (DIBH). This study quantifies the gross target volume (GTV) motion on cine-MRI of apical lung lesions undergoing a SBRT in a MR-Linac and supports the patient specific treatment gating pre-selection.

**Material and methods:**

A total of 12 patients were retrospectively enrolled in this study. During simulation and treatment fractions, sagittal 0.35 T cine-MRI allows real-time GTV motion tracking. Cine-MRI has been exported, and an in-house developed MATLAB script performed image segmentation for measuring GTV centroid position on cine-MRI frames. Motion measurements were performed during the deep inspiration phase of DIBH patient and during all the session for FB patient. Treatment plans of FB patients were reoptimized using the same cost function, choosing the 3 mm GTV-PTV margin used for DIBH patients instead of the original 5 mm margin, comparing GTV and OARs DVH for the different TP.

**Results:**

GTV centroid motion is <2.2 mm in the antero-posterior and cranio-caudal direction in DIBH. For FB patients, GTV motion is lower than 1.7 mm, and motion during the treatment was always in agreement with the one measured during the simulation. No differences have been observed in GTV coverage between the TP with 3-mm and 5-mm margins. Using a 3-mm margin, the mean reduction in the chest wall and trachea–bronchus Dmax was 2.5 Gy and 3.0 Gy, respectively, and a reduction of 1.0 Gy, 0.6 Gy, and 2.3% in Dmax, Dmean, and V5Gy, respectively, of the homolateral lung and 1.7 Gy in the contralateral lung Dmax.

**Discussions:**

Cine-MRI allows to select FB lung patients when GTV motion is <2 mm. The use of narrower PTV margins reduces OARs dose and maintains target coverage.

## 1 Introduction

Stereotactic body radiation therapy (SBRT) is currently considered the standard of care for early-stage inoperable non-small cell lung cancer (NSCLC) (1, 2), and there is growing evidence for its applications in the oligometastatic disease setting (3). Due to the high dose per fraction and the sharp dose gradients delivered in SBRT, accurate image guidance is required to ensure an effective and safe treatment delivery. The state-of-the-art in-room image guidance radiotherapy (IGRT) systems (4) generally provide the tumor position verification just before the treatment.

During the treatment delivery, most IGRT systems prevent target misplacement but do not effectively manage intra-fraction motion, unless the treatment system is tracking the tumor. Intra-fraction motion management is therefore still a major challenge in SBRT of lung lesions, also considering that intra-fraction position variability correlates with treatment delivery time (5).

Different motion management strategies have been developed, especially for the determination of ITV (6): such approaches assure target coverage but increase the volume of irradiated healthy tissues. Optimal results can be achieved by double-arc VMAT delivery technique in terms of dose distribution, also reducing treatment delivery time in comparison with non-coplanar IMRT beams (7).

Even though the dose conformality is achievable within the mentioned technologies, intra-fraction motion management may be not efficient, even using surrogate motion system (8). On-board magnetic resonance imaging (MRI) allows the non-invasive continuous and direct monitoring of target and organs at risk (OARs) through the acquisition of 4D-MRI and 2-D cine-MRI during the entire RT course, introducing an unprecedented significant innovation in the field of radiation oncology (9, 10).

The introduction of MRI-Linacs into clinical practice has brought new approaches for the intra- and inter-fraction motion management (11, 12). On-line imaging allows to directly track structures, as target or OARs, on the cine-MRI (13) and to perform active direct beam gating, stopping the radiation beam when a structure moves beyond a user defined tolerance region called boundary. Different strategies can be used in boundary definition (14):

- Direct gross target volume (GTV) tracking: the boundary can be set as the PTV or an isodose level.
- Indirect tracking: a surrogate structure that moves integral to the GTV is used for the gating.

This development led to a preference for the use of direct/indirect gating approach instead of ITV-based motion management.

Previous to the delivery, this hybrid system allows a daily plan dose distribution optimization based on the daily anatomy of the patients (15), employing a different approach to the planning to enhance the online treatment adaptation (16, 17), in terms of accuracy and computational time.

Lung treatments can be delivered in FB or using different breathing modality such as the DIBH that can be performed at different respiratory phases (18). However, using an MR-Linac, whichever mode is chosen, direct beam gating can always be performed by tracking the GTV on the cine-MRI. Another peculiarity of MR-Linacs is that it is possible to perform beam gating without the need to use devices like spirometer, fiducials, or abdominal belt to trigger the beam off.

The use of DIBH treatment for lung lesions can reduce the total irradiated lung volume and dose to near OARs with respect to free breathing (FB) treatment (19–21) and can be successfully customized for the different clinical scenarios.

During treatment MRI simulation, the breathing modality is evaluated to identify the most appropriate gating delivery technique, based on both lesion’s and patient’s factors compliance (i.e., location, dimensions, general conditions, and compliance). However, also the most recent guidelines (22) do not suggest a quantitative method for the selection of the best gating strategy (FB or DIBH) for patient treated on an MR-Linac with cine-MRI-based gating.

Especially for DIBH treatment, patient’s compliance is a key point to prevent exhausting treatment sessions with sub-optimal dose delivery (23). As an example, apical lung lesions, especially the most central ones, generally present less motion amplitude in the right–left (RL) direction with respect to cranio-caudal (CC) and antero-posterior (AP) direction and present less motion during the breathing cycle with respect to lower lobe lesions (24–28). However, DIBH treatments turn out to be longer and more demanding for the patient and should therefore be carefully evaluated by the attending physician against other delivery settings (i.e., FB) (29).

The aim of this study is to provide a quantitative method for patient treatment selection between DIBH and FB, based on GTV centroid motion measurements performed on the simulation 0.35 T cine-MRI (30) and then evaluate the GTV to PTV margins reduction in the case of low mobility targets treated in FB (31).

## 2 Materials and methods

### 2.1 Patient selection and images dataset

A total of 12 patients with apical lung tumor who underwent MRI-guided SBRT have been retrospectively enrolled in this retrospective single-center study. All treatments were performed on MRIdian hybrid system (ViewRay Inc., Mountain View, CA) that combines a 0.35 T on board MRI scanner with a 6-MV flattening filter free (FFF) Linac system (32).

All patients included in the study underwent a simulation session where different images set were acquired sequentially. First, an MRI simulation session was performed directly inside the MR-Linac where different 3D MRI and cine-MRI were acquired:

- Two 3D MRI scans were performed with a true fast imaging with steady-state precession (TRUFI) sequence with image resolution of 1.5 × 1.5 × 3 mm^3^ and 1.5 × 1.5 × 1.5 mm^3^ and an acquisition time of 25 and 172 s, respectively. The former was performed under DIBH, the latter in FB. With the patient still inside the MR-Linac, a radiation oncologist had contoured the GTV on MR images to further allow GTV tracking on the subsequent cine-MRI.
- RTT sets the TDS tracking algorithm in order to have real-time GTV tracking on the cine-MRI, and then, two sagittal 2D cine TrueFISP sequence (true fast imaging with steady state precession) were recorded with a spatial resolution of 0.35 × 0.5 cm^2^ and an acquisition frequency of 4 frames/s:
  - a cine-MRI with at least 20 s of FB
  - a cine-MRI with several DIBH.

During the DIBH cine-MRI, a multidisciplinary team, composed of a radiation oncologist, a physicist, and an RTT, had verified the clinical/dosimetric suitability and the patient’s compliance and had define the treatment gating techniques. However, the choice between the two different gating strategies (FB and DIBH) was performed without a quantitative analysis on the GTV motion.

Next to the MRI simulation session, a CT simulation session was carried out (GE, Optima CT580 W, HiSpeed Dx/I Spiral): 1.25 mm slice thickness, without contrast agent, acquired in the same MRI simulation position, and with the same positioning system. The CT image was acquired in DIBH or FB, based on the information obtained during the MRI simulation. CT images are needed only for the creation of the electronic density map of the patient. CT image was then deformably fused to the MRI images to perform the nominal dose distribution that will be the reference dose distribution during the first treatment fraction. In case of a daily anatomical variation, the nominal dose distribution is online re-optimized to achieve optimal target coverage and OARs sparing.

The acquisition in DIBH is carried out without the aid of dedicated breath hold systems (i.e., RPM, surface surrogate, …); in fact, thanks to the possibility of performing cine-MRI, the TDS can directly track the target on the cine images in real time and perform the beam gating.

Six (50%) out of the 12 selected patients were treated in FB and six patients in DIBH gating delivery technique. All patients’ fractionation schemes are listed in **Table 1**.

**Table 1:** Numbers of fractions for the FB and DIBH patients and dose prescribed to the isodose level.

Patient ID	Gating	Fractions	Prescription
1	FB	5	50 Gy@80%
2	FB	5	50 Gy@80%
3	FB	3	42 Gy@80%
4	FB	5	50 Gy@80%
5	FB	5	50 Gy@80%
6	FB	5	40 Gy@80%
7	DIBH	8	40 Gy@50%
8	DIBH	5	40 Gy@80%
9	DIBH	8	56 Gy@50%
10	DIBH	5	50 Gy@80%
11	DIBH	5	50 Gy@80%
12	DIBH	5	50 Gy@80%

Intensity-modulated radiation therapy (IMRT) step-and-shoot treatment plans were then calculated using the MRIdian treatment planning system (TPS). Dose calculation was carried out with the Monte Carlo algorithm on the MRIdian TPS (2,500,000 histories) using a calculation grid of 0.2 cm × 0.2 cm × 0.2 cm.

When optimizing the treatment plan, we pay special attention to the total treatment time, which we always keep below 13 min by adjusting the optimization parameters. For FB treatments, this is the actual treatment time. For DIBH treatments, we need to add the gating time to this. The gating time is closely linked to the patient’s compliance and therefore varies from case to case.

In the clinical practice, PTV was created through an isotropic GTV expansion with a 0.3-cm margin for all DIBH treatments and 0.5 cm expansion for all FB treatments. GTV was set as direct gating structure, and the gating boundary was set equal to PTV or to the 95% isodose level. The TDS had been set in order to stop the dose delivery when more than 5% of GTV was outside the boundary for both FB and DIBH treatments, according to internal department guidelines. The mentioned values have been user defined and reflect the clinical experience of our center since 2017; however, they had been borrowed from clinical practice on conventional linac. The absence of international guidelines for MRgRT treatments has resulted in many practices common in treatments with traditional linacs being carried over to MR-Linacs. The 3-mm margin used for DIBH treatments is considered to be the lowest possible due to other limitations like image and dose grid resolution.

### 2.2 Image segmentation

**Figure 1:**
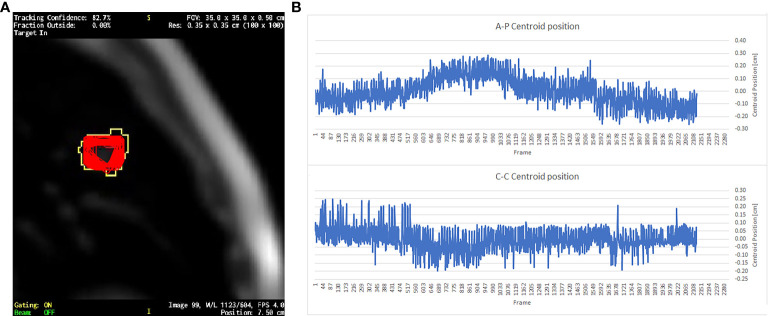
**(A)** A single frame of a sagittal cine-MRI where the red contours represent the superposition of the GTV contours exported from all the 2,124 cine frames; yellow contour is the gating boundary. **(B)** The GTV centroid relative position respect to its mean value in the AP direction and CC direction.

ViewRay tracking algorithm overwrites over the grayscale image of the cine-MRI the gating boundary in yellow contour and the outline of the structure to be tracked (in this case GTV) in red (**Figure 1**). GTV contour was tracked for beam gating on all patients (both DIBH and FB) analyzed in this study. Positional data of the tracked structures over the cine-MRI could not be exported from the TPS, but it was possible to export the cine-MRI video in mp4 format. Employing an in-house developed MATLAB® R2019a (The Math-Works, Inc., Natick, MA) script, it was possible to identify the GTV contour and measure the centroid position. In the mp4 video, the only two colors over the grayscale of the MRI were the yellow of the gating boundary and the red of the GTV. All the frames of the mp4 video were exported as single RGB images where it was possible to find a threshold in the values of the RGB channels to select only the red GTV contour or the yellow outline of the gating boundary. In this way, it was therefore possible to select only the GTV structure to measure the position of the GTV centroid on every cine-MRI frames.

### 2.3 Motion analysis

The target’s motion was evaluated by measuring the standard deviation of the GTV centroid position (**Figure 1**). The motion was measured in CC and AP directions, which were the only two directions visible on the sagittal cine-MRI (**Figure 1**). However, it can be assumed that the movement in the third direction of RL is of the same order of magnitude as the others (24, 25).

Motion analyses were performed on the simulation cine-MRI and over all the cine-MRI recorded during the treatment fractions for all the patients.

Up to four tracking points can be manually placed on the MRI images during cine-MRI acquisition, which the clinical algorithm can track together with the contour of the GTV. Coordinates of such tracking points on all the cine frames can then be exported from the TPS. These values can be used to compare the values obtained by in-house tracking algorithm, in order to evaluate its accuracy. As a case test, the five fractions of FB patient no. 6 were selected, placing manually a tracking point at the center of the GTV as a benchmark value to be compared with the centroid position obtained by the homemade algorithm. This was done only on a single patient because this is a retrospective study and tracking points were placed at the time of the treatment only on this patient.

Different measurements of GTV motion were performed on DIBH patients:

- Simulation cine-MRI GTV motion measurements:
  - in deep inspiration phase (DIP)
  - in free breathing phase (FBP)
- Treatment cine-MRI GTV motion measurements:
  - in DIP

At least six complete deep inspirations cycle were available for each treatment cine in DIBH patients.

For FB patients, GTV motion was measured on all frames of each cine separately:

- in simulation cine-MRI
- in treatment fractions cine-MRI
- mean GTV motion measured for each patient

Different comparisons between the measured GTV motion were considered in this study:

- DIBH patients:
  - DIP vs. FBP GTV motion
  - DIP GTV motion simulation vs. first fraction vs. last fraction
- FB patients:
  - simulation vs. treatment motion
  - mean GTV motion vs. DIP GTV motion

Homolateral lung and GTV volume had been also considered, together with the movement of the hepatic dome, to further evaluate possible correlation with GTV CC motion on FB patients using the Pearson correlation coefficient.

### 2.4 Dosimetric assessment of margin reduction

The dosimetric effect of reducing the GTV to PTV margin was evaluated on FB patients, comparing dose–volume constraints of the nearest OARs in treatment plans optimized with different GTV to PTV margins. In particular, two different margins were evaluated:

- standard margin (SM): GTV to PTV equal to 5 mm, which is the current treatment margin used for FB patients
- magnetic resonance image guided margin (MRIgM): GTV to PTV equal to 3 mm, which is the current treatment margin used for DIBH patients.

Treatment plans with the described margins were generated for the different margins using the same cost function. Maximum dose (Dmax) of the contralateral lung, chest wall, and trachea–bronchus structures were compared, together with mean dose (Dmean) and V5Gy of both lungs and V20Gy of the homolateral lung. PTV and GTV coverage were also considered in the evaluation of margin reduction comparing V100% and V80% of the prescribed dose in both structures for the different margins.

## 3 Results

A total of 77 cine-MRI had been exported, for a total of more than 13 h of recorded cine-MRI and more than 190,000 frames.

GTV centroid standard deviations for all the cine-MRI measured for FB patients are shown in **Table 2**, for both directions of motion visible on the cine-MRI. Observed motions for FB patients range from 0.04 cm to 0.17 cm in AP and CC directions, while mean motion during all the treatment goes from 0.07 cm to 0.12 cm in the AP direction and from 0.06 cm to 0.12 cm in the CC direction for all the FB patients.

**Table 2:** GTV motion defined as standard deviation of the centroid position, measured during the simulation (SIM) and the mean, minimum, and maximum motion measured during the treatment fractions for FB patients and DIP of DIBH patients in the two directions visible on the cine-MRI.

Patient	AP [cm]			CC [cm]		
	SIM	Mean SD	Min–Max SD	SIM	Mean SD	Min–Max SD
1	0.06	0.09	0.06–0.11	0.11	0.09	0.07–0.12
2	0.15	0.10	0.08–0.15	0.10	0.10	0.07–0.14
3	0.13	0.12	0.07–0.17	0.06	0.06	0.04–0.08
4	0.06	0.07	0.06–0.08	0.08	0.06	0.06–0.08
5	0.10	0.09	0.05–0.12	0.17	0.12	0.05–0.17
6	0.10	0.08	0.04–0.11	0.11	0.08	0.05–0.11
7	0.07	0.08	0.04–0.13	0.04	0.11	0.04–0.20
8	0.07	0.09	0.06–0.12	0.07	0.08	0.04–0.12
9	0.09	0.08	0.04–0.12	0.13	0.09	0.06–0.13
10	0.06	0.09	0.04–0.11	0.05	0.11	0.05–0.22
11	0.08	0.11	0.08–0.17	0.06	0.14	0.06–0.21
12	0.15	0.13	0.07–0.18	0.12	0.12	0.09–0.17

In all fractions of patient no. 6, where a tracking point was manually placed at the center of the GTV, the standard deviation of the motion of the tracking point, averaged over all the fractions, is 0.07 cm in AP direction and 0.08 cm in CC, in agreement with the ones measured with the in-house script.

**Figure 2:**
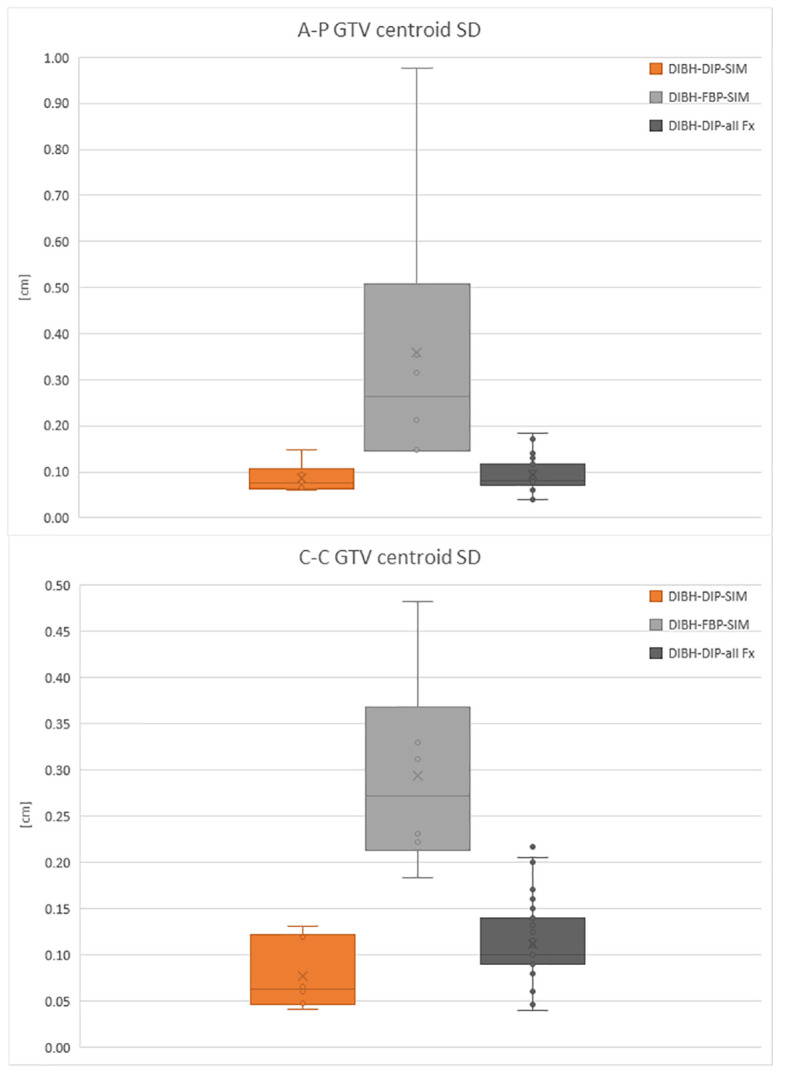
Box plot of the GTV centroid SD in antero-posterior (A-P) direction and cranio-caudal (C-C) direction for DIBH patients in the DIP.

**Figure 2** reports a box plot of DIBH patients’ motion, analyzed both during the DIP, where GTV centroid motion ranges from 0.04 cm to 0.13 cm in both directions during the simulation and from 0.04 cm to 0.22 cm during the treatment fractions. In the FBP acquired during the simulation, the motion is between 0.14 cm and 0.98 cm in both directions. In the box plot, the horizontal lines represent the median and the 25th and 75th percentile, while the whiskers represent the minimum and maximum value, excluding the outliers represented as points outside the whiskers.

**Figure 3:**
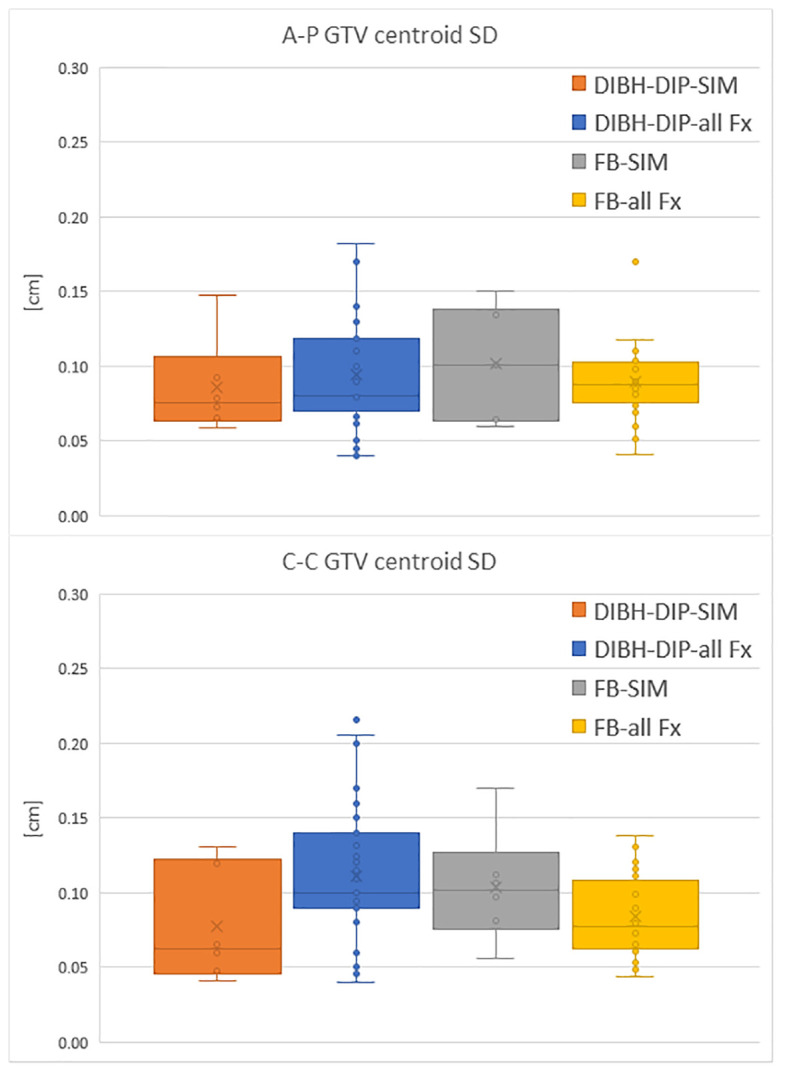
Comparison of the GTV centroid SD in A-P direction and C-C direction between DIP of DIBH patients and motion measured on FB patients. Motion is evaluated both during the simulation (SIM) and the treatment fractions (all Fx).

FB patients’ GTV motions recorded in simulation cine-MRI are shown in **Figure 3** together with the GTV motion measured on all the FB cine-MRI and the GTV motion evaluated during the DIP in DIBH patients.

Possible correlations between the amplitude of GTV CC motion and the GTV volume, the ratio between GTV and lung volumes, and the hepatic dome motion were investigated; **Table 3** shows the GTV motions versus the volume of GTV and homolateral lung contours and the maximum amplitude of hepatic dome motion in CC direction. In **Table 3**, the ratio between the GTV volume and the lung volume is also presented.

**Table 3:** Hepatic dome motion compared with GTV and lung properties.

Patient	GTV motion [cm]	V lung [cc]	V GTV [cc]	V GTV/V lung [×10^−4^]	Hepatic dome motion [cm]
1	0.09	1,934.39	1.92	9.93	1.68
2	0.10	1,447.68	0.47	3.25	1.36
3	0.06	2,301.69	1.86	8.08	1.28
4	0.06	2,395.99	8.95	37.35	2.00
5	0.12	2,764.78	0.42	1.52	1.60
6	0.08	2,132.50	36.49	171.11	2.60

The measured Pearson correlation coefficient between the GTV CC motion and the GTV volume, the ratio between GTV and lung volume, and the hepatic dome motion are, respectively, −0.25, −0.24, and −0.18.

Dosimetric differences between the treatment plans generated with SM and MRIgM can be found in **Table 4** (MRIgM-SM).

**Table 4:** DVH differences between treatment plans generated with magnetic resonance image guided margin (MRIgM) and standard margin (SM).

Structure	DVH index	Patient						
		1	2	3	4	5	6	mean
PTV	V80%	1.00%	0.00%	5.70%	5.54%	0.09%	3.98%	2.72%
	V100%	5.01%	9.11%	12.97%	15.58%	6.88%	3.37%	8.82%
GTV	V80%	0.00%	0.00%	0.00%	0.03%	0.00%	−0.16%	−0.02%
	V100% [%]	−14.17%	−13.61%	4.98%	20.58%	−6.20%	−2.84%	−1.88%
	V100% [cc]	−0.27	−0.07	−0.26	−1.27	−0.06	−5.17	−1.18
Homolateral lung—GTV	V20Gy	−1.83%	−1.67%	−0.83%	−1.66%	−0.64%	−0.22%	−1.14%
	D_mean_ (Gy)	−0.91	−0.78	−0.35	−0.71	−0.57	−0.01	−0.56
	V5Gy	−3.80%	−3.34%	−1.74%	−2.25%	−2.74%	−0.32%	−2.37%
Contralateral lung	D_max_ (Gy)	−0.65	−1.27	−0.36	−2.92	−4.14	−1.02	−1.73
	D_mean_ (Gy)	−0.18	−0.01	−0.13	−0.14	−0.34	−0.29	−0.18
	V5Gy	−0.03%	−0.85%	−0.19%	−1.37%	−1.52%	−2.44%	−1.07%
Chest wall	D_max_ (Gy)	−5.10	−3.73	−0.70	−1.82	−3.39	−0.56	−2.55
Trachea and bronchial tree	D_max_ (Gy)	−6.85	−2.57	−1.85	−0.03	−5.82	−0.88	−3.00

The average difference over all patients is reported in the last column of **Table 4**. A mean Dmax reduction of 2.5 Gy and 3.0 Gy is observed in the chest wall and trachea–bronchus, respectively, with reductions of more than 5 Gy on a single patient when narrower margins are used.

Dmean and V5Gy decrease in both lungs using MRIgM together with V20Gy of the homolateral lung.

As expected, the 80% isodose covers the whole volume of the GTV using both treatment margins, while there is an increase in PTV V80% and V100% of treatment plans with MRIgM. A mean reduction of 1.88% in GTV V100% is observed when using MRIgM as expected if the treatment plan is being delivered to a smaller volume.

## 4 Discussion

The movement of the GTV as a result of the respiratory cycle is not homogeneous in the different parts of the lung, as has already been described in the literature (26–28). This study had investigated the possibility of treating patients with a FB technique to increase patient’s compliance and reduce treatment time respect to DIBH treatments in the case of upper lobe lesions. Sagittal cine-MRI were used to measure GTV centroid motion in AP and CC directions.

The motion measured during DIP of DIBH patients was comparable with the one observed by Van Sornsen de Koste et al. (30) with a similar method based on GTV centroid tracking on cine-MRI. The Motions measured in other works are difficult to compare because of different lesion positions inside the lungs or different gating and imaging techniques, but overall motion appeared to be comparable with the one measured by Barnes et al. (19) and Britton et al. (25), considering only upper lung lesions data of these works.

No correlations were found between GTV CC motion and hepatic dome motion, GTV volume, and the ratio between GTV and lung motion.

Analysis of GTV motion performed on both FB and DIBH patients had shown that the GTV motion observed during simulation was representative of the motion that will be present throughout the treatment, for FB patients. Indeed, based on the collected data, the motion measured during simulation was always in agreement with the one observed during treatment fractions (**Figure 3**).

This result supports the choice to perform treatment in FB in those patients who present during the MRI simulation a motion amplitude lower than 2 mm. When compared to DIBH patients, the motion of the GTV during treatment of FB patients, which have a GTV motion smaller than 2 mm in simulation, turned out to be similar than that observed on DIBH patients during the DIP (as shown in **Figure 3**).

This further justifies the choice of the GTV to PTV margin of 3 mm, keeping in mind that the volume of the GTV on the MRI acquired in FB already turns out to be an average of the position of the GTV over the 172 s of image acquisition time (33). This point also appears in the simulation CT where the structures can be also contoured for the treatment planning.

It was also possible to measure GTV motion manually placing tracking points on the simulation and treatment images, thanks to a dedicated functionality of the ViewRay TPS. This procedure had been performed on a patient treated in FB: a tracking point was placed in the center of the GTV to obtain an additional measure of GTV motion in order to validate the in-house script used for image segmentation and motion tracking. The standard deviation of the position of the tracking points turned out to be in agreement with the motion of the GTV analyzed using our technique (0.07 cm in AP direction and 0.08 cm in CC).

When GTV motion is >2 mm in at least one of the two directions during the MRI FB simulation, the use of DIBH gating technique leads to a considerable reduction in GTV motion as observed in **Figure 2** for the patient treated in DIBH, which then justifies the use of a more demanding active gating technique for the patient and an increased treatment time.

The main advantages of FB treatments are a reduction in treatment time and less patient fatigue from holding their breath, and a reduction in the time spent in an uncomfortable position in the MR-Linac bore (arms raised above the head throughout the treatment).

Finally, the possibility to perform simulation cine-MRI and gating during treatment on this hybrid system safely allows to perform a margin reduction, since the GTV segmentation on the simulation and treatment cine-MRI is performed by the same algorithm, which also feeds the treatment gating. Another well-known advantage of gating treatment is the dose reduction in the surrounding healthy tissue, since no dose would be delivered if the GTV motion resulted greater than the boundary during treatment. On the other hand, for linacs without integrated MRI, simulation cine-MRI can always be performed if the facility is equipped with a dedicated MRI. Therefore, cine-MRI could be clinically used to assess lesion motion; however, the GTV segmentation software on the cine-MRI could be different from that used for gating, potentially introducing an additional source of error. In order to better explore such workflow, further studies are required.

The reduction in treatment margins in the planning stage leads to a reduction in OARs dose. In particular, in the case of OARs located near or even inside the PTV, the reduction in GTV to PTV margins obviously increases the distance for the dose fall-off outside the target, increasing PTV coverage and decreasing the dose to OARs. A reduction in the GTV V100% was observed in treatment plans with MRIgM; however, it was still clinically acceptable. Computing the treatment plans with MRIgM, the same cost function had been employed to exclude differences due to the optimizer; however, a finer optimization could lead to treatment plans with the same GTV V100%.

The impact of this work on SABR of the lung is expressed in the fact that we can identify a target movement threshold to determine which patients would benefit from DIBH, a more stressful technique. With a view to margin reduction, this work can be a first step toward realizing personalized treatment margins for FB patients as well.

A limitation of this study is the lack of information about the motion in the right-left (RL) direction; however, the motion in this direction is lower or of the same amplitude of the one observed on AP and CC directions [16,17]. The ongoing release of the new MRIdian treatment delivery system will permit recording cine-MRI in the three directions, tracking different regions of interest (both direct and indirect tracking). Therefore, future studies will also evaluate motion in the RL direction.

Further analysis is ongoing to complete an overall 3D GTV lung lesions movement mapping: this would provide a clinical decision tool to select the optimal gating strategy and GTV to PTV margins.

This study suggests a methodology to define MRIgRT SBRT for apical lung lesions. During MRI simulation, a motion of the GTV of <2 mm in all examinable directions support the possibility of FB treatment. Since GTV motion in FB can be compared with treatment in DIBH, the same treatment margins can be used, improving patient compliance, reducing both treatment time and dose to surrounding OARs, without compromising target coverage.

## Data availability statement

The original contributions presented in the study are included in the article/supplementary material. Further inquiries can be directed to the corresponding author.

## Ethics statement

Ethical approval was not required for the study involving humans in accordance with the local legislation and institutional requirements. Written informed consent to participate in this study was not required from the participants or the participants’ legal guardians/next of kin in accordance with the national legislation and the institutional requirements.

## Author contributions

MG: Conceptualization, Data curation, Formal Analysis, Investigation, Methodology, Software, Writing – original draft, Writing – review & editing. MN: Conceptualization, Investigation, Supervision, Visualization, Writing – review & editing. AC: Supervision, Validation, Visualization, Writing – review & editing. GM: Supervision, Validation, Visualization, Writing – review & editing. DC: Validation, Visualization, Writing – review & editing. LB: Supervision, Validation, Visualization, Writing – review & editing. GC: Supervision, Validation, Visualization, Writing – review & editing. AR: Supervision, Validation, Visualization, Writing – review & editing. CV: Conceptualization, Data curation, Supervision, Validation, Visualization, Writing – review & editing. MAG: Funding acquisition, Visualization, Writing – review & editing. LI: Funding acquisition, Visualization, Writing – review & editing. LP: Conceptualization, Investigation, Methodology, Project administration, Supervision, Validation, Visualization, Writing – review & editing.

## References

[1] Grills IS Hope AJ Guckenberger M Kestin LL Werner-Wasik M Yan D. A collaborative analysis of stereotactic lung radiotherapy outcomes for early-stage non-small-cell lung cancer using daily online cone-beam computed tomography image-guided radiotherapy. J Thorac Oncol (2012) 7:1382–93. doi: 10.1097/JTO.0b013e318260e00d 22843086

[2] Baumann P Nyman J Hoyer M Wennberg B Gagliardi G Lax I. Outcome in a prospective phase II trial of medically inoperable stage I non-small-cell lung cancer patients treated with stereotactic body radiotherapy. J Clin Oncol (2009) 27:3290–6. doi: 10.1200/JCO.2008.21.5681 19414667

[3] Milano MT Philip A Okunieff P. Analysis of patients with oligometastases undergoing two or more curative-intent stereotactic radiotherapy courses. Int J Radiat Oncol Biol Phys (2009) 73:832–7. doi: 10.1016/j.ijrobp.2008.04.073 18760543

[4] Dieterich S Green O Booth J. SBRT targets that move with respiration. Physica Med (2018) 56:19–24. doi: 10.1016/j.ejmp.2018.10.021 30527085

[5] Purdie TG Bissonnette JP Franks K Bezjak A Payne D Sie F. Cone-beam computed tomography for on-line image guidance of lung stereotactic radiotherapy: localization, verification, and intrafraction tumor position. Int J Radiat Oncol Biol Phys (2007) 68:243–52. doi: 10.1016/j.ijrobp.2006.12.022 17331671

[6] Korreman SS. Image-guided radiotherapy and motion management in lung cancer. Br J Radiol (2015) 88. doi: 10.1259/bjr.20150100 PMC4628536 25955231

[7] Xhaferllari I El-Sherif O Gaede S. Comprehensive dosimetric planning comparison for early-stage, non-small cell lung cancer with SABR: Fixed-beam IMRT versus VMAT versus TomoTherapy. J Appl Clin Med Phys (2016) 17:329–40. doi: 10.1120/jacmp.v17i5.6291 PMC5874107 27685129

[8] Brandner ED Chetty IJ Giaddui TG Xiao Y Huq MS. Motion management strategies and technical issues associated with stereotactic body radiotherapy of thoracic and upper abdominal tumors: A review from NRG oncology. Med Phys (2017) 44:2595–612. doi: 10.1002/mp.12227 PMC5473359 28317123

[9] Corradini S Alongi F Andratschke N Belka C Boldrini L Cellini F. MR-guidance in clinical reality: Current treatment challenges and future perspectives. Radiat Oncol (2019) 14:1–22. doi: 10.1186/s13014-019-1308-y PMC6551911 31167658

[10] Ménard C van der Heide UA. Introduction: magnetic resonance imaging comes of age in radiation oncology. Semin Radiat Oncol (2014) 24:149–50. doi: 10.1016/j.semradonc.2014.02.001 24931084

[11] Cusumano D Dhont J Boldrini L Chiloiro G Teodoli S Massaccesi M. Predicting tumour motion during the whole radiotherapy treatment: a systematic approach for thoracic and abdominal lesions based on real time MR. Radiother Oncol (2018) 129:456–62. doi: 10.1016/j.radonc.2018.07.025 30144955

[12] Thomas DH Santhanam A Kishan A Cao M Lamb J Min Y. Initial clinical observations of intra-and interfractional motion variation in MR-guided lung SBRT. British J Radiol (2018) 91. doi: 10.1259/bjr.20170522 PMC5965474 29166129

[13] Dhont J Vandemeulebroucke J Cusumano D Boldrini L Cellini F Valentini V. Multi-object tracking in MRI-guided radiotherapy using the tracking-learning-detection framework. Radiother Oncol (2019) 138:25–9. doi: 10.1016/j.radonc.2019.05.008 31136959

[14] Votta C Cusumano D Boldrini L Dinapoli N Placidi L Turco G. Delivery of online adaptive magnetic resonance guided radiotherapy based on isodose boundaries. Phys Imaging Radiat Oncol (2021) 18:78–81. doi: 10.1016/j.phro.2021.05.005 34258412 PMC8254198

[15] Placidi L Romano A Chiloiro G Cusumano D Boldrini L Cellini F. On-line adaptive MR guided radiotherapy for locally advanced pancreatic cancer: Clinical and dosimetric considerations. Tech Innov Patient Support Radiat Oncol (2020) 15:15–21. doi: 10.1016/j.tipsro.2020.06.001 32642565 PMC7334416

[16] Placidi L Nardini M Cusumano D Boldrini L Chiloiro G Romano A. VMAT-like plans for magnetic resonance guided radiotherapy: Addressing unmet needs. Physica Med (2021) 85:72–8. doi: 10.1016/j.ejmp.2021.05.002 33979726

[17] Nardini M Placidi L. Robust online adaptive planning: Toward a uniform MR-LINAC treatment planning technique. Advances in Magnetic Resonance Technology and Applications (2022) 8:101–22. doi: 10.1016/B978-0-323-91689-9.00025-X

[18] Botticella A Levy A Auzac G Chabert I Berthold C Le Pechoux C. Tumour motion management in lung cancer: A narrative review. Transl Lung Cancer Res (2021) 10:2011–7. doi: 10.21037/tlcr-20-856 PMC8107759 34012810

[19] Barnes EA Murray BR Robinson DM Underwood LJ Hanson J Roa WHY. Dosimetric evaluation of lung tumor immobilization using breath hold at deep inspiration. Int J Radiat Oncol Biol Phys (2001) 50(4):1091–8. doi: 10.1016/s0360-3016(01)01592-9 11429237

[20] Kim DJW Murray BR Halperin R Roa WHY. Held-breath self-gating technique for radiotherapy of non-small-cell lung cancer: a feasibility study. Int J Radiat Oncol Biol Phys (2001) 49(1):43–9. doi: 10.1016/s0360-3016(00)01372-9 11163496

[21] Fjellanger K Rossi L Heijmen BJM Pettersen HES Sandvik IM Breedveld S. Patient selection, inter-fraction plan robustness and reduction of toxicity risk with deep inspiration breath hold in intensity-modulated radiotherapy of locally advanced non-small cell lung cancer. Front Oncol (2022) 12:966134. doi: 10.3389/fonc.2022.966134 36110942 PMC9469652

[22] Aznar MC carrasco de fez P Corradini S Mast M McNair H Meattini I. ESTRO-ACROP guideline: Recommendations on implementation of breath-hold techniques in radiotherapy. Radiother Oncol (2023) 185. doi: 10.1016/j.radonc.2023.109734 37301263

[23] Placidi L Cusumano D Boldrini L Votta C Pollutri V Antonelli MV. Quantitative analysis of MRI-guided radiotherapy treatment process time for tumor real-time gating efficiency. J Appl Clin Med Phys (2020) 21:70–9. doi: 10.1002/acm2.13030 PMC7701108 33089954

[24] Seppenwoolde Y Shirato H Kitamura K Shimizu S Van Herk M Lebesque JV. Precise and real-time measurement of 3D tumor motion in lung due to breathing and heartbeat, measured during radiotherapy. Int J Radiat Oncol Biol Phys (2002) 53(4):822–34. doi: 10.1016/s0360-3016(02)02803-1 12095547

[25] Britton KR Starkschall G Tucker SL Pan T Nelson C Chang JY. Assessment of gross tumor volume regression and motion changes during radiotherapy for non-small-cell lung cancer as measured by four-dimensional computed tomography. Int J Radiat Oncol Biol Phys (2007) 68:1036–46. doi: 10.1016/j.ijrobp.2007.01.021 17379442

[26] Rabe M Thieke C Düsberg M Neppl S Gerum S Reiner M. Real-time 4DMRI-based internal target volume definition for moving lung tumors. Med Phys (2020) 47:1431–42. doi: 10.1002/mp.14023 31955430

[27] Redmond KJ Song DY Fox JL Zhou J Rosenzweig CN Ford E. Respiratory motion changes of lung tumors over the course of radiation therapy based on respiration-correlated four-dimensional computed tomography scans. Int J Radiat Oncol Biol Phys (2009) 75:1605–12. doi: 10.1016/j.ijrobp.2009.05.024 19931739

[28] Yu ZH Lin SH Balter P Zhang L Dong L. A comparison of tumor motion characteristics between early stage and locally advanced stage lung cancers. Radiother Oncol (2012) 104:33–8. doi: 10.1016/j.radonc.2012.04.010 22677039

[29] Keall PJ Mageras GS Balter JM Emery RS Forster KM Jiang SB. The management of respiratory motion in radiation oncology report of AAPM Task Group 76. Med Phys (2006) 33:3874–900. doi: 10.1118/1.2349696 17089851

[30] van Sörnsen de Koste JR Palacios MA Bruynzeel AME Slotman BJ Senan S Lagerwaard FJ. MR-guided gated stereotactic radiation therapy delivery for lung, adrenal, and pancreatic tumors: A geometric analysis. Int J Radiat Oncol Biol Phys (2018) 102:858–66. doi: 10.1016/j.ijrobp.2018.05.048 30061007

[31] Masuda H Kawahara D Saito A Kimura T Ozawa S Nakashima T. Reduction of margin to compensate the respiratory tumor motion by the analysis of dosimetric internal target volume in lung SBRT with nonuniform volume prescription method. Med Phys (2021) 48:3200–7. doi: 10.1002/mp.14871 33792065

[32] Mutic S Dempsey JF. The viewRay system: magnetic resonance-guided and controlled radiotherapy. Semin Radiat Oncol (2014) 24:196–9. doi: 10.1016/j.semradonc.2014.02.008 24931092

[33] Wolthaus JWH Sonke JJ van Herk M Belderbos JSA Rossi MMG Lebesque JV. Comparison of different strategies to use four-dimensional computed tomography in treatment planning for lung cancer patients. Int J Radiat Oncol Biol Phys (2008) 70:1229–38. doi: 10.1016/j.ijrobp.2007.11.042 18313530

